# Angioleiomyoma of the Hand: A Report of Two Cases

**DOI:** 10.7759/cureus.36016

**Published:** 2023-03-11

**Authors:** Wendolin J Ortiz, John J Eager, Mario Cervantes

**Affiliations:** 1 General Surgery, Universidad Autónoma de Baja California, Mexicali, MEX; 2 Pathology, Hospital Corporation of America (HCA) Houston Healthcare West, Houston, USA; 3 Pathology, Hospital Corporation of America (HCA) Houston Healthcare Pearland, Houston, USA; 4 Orthopaedic Surgery, Hospital Corporation of America (HCA) Houston Healthcare West, Houston, USA

**Keywords:** biopsy, hand tumor, vascular leiomyoma, ct scan, angioleiomyoma

## Abstract

We present a case series of two rare cases of angioleiomyoma of the hand, an infrequent type of benign tumor. In the first case, a 41-year-old female presented with a left thumb mass that had increased in size over two years. Imaging studies revealed a destructive lesion involving the first webspace with infiltration of the first metacarpal, and the mass was initially suspected to be a sarcoma. However, a percutaneous image-guided fine-needle aspiration (US-FNA) and ultrasound-guided core-needle biopsy (USG-CNB) of the left-hand mass confirmed the diagnosis of angioleiomyoma. The mass was surgically excised, and the final diagnosis was consistent with the earlier USG-CNB. In the second case, a 63-year-old man with end-stage renal disease presented for consultation regarding dialysis access creation. During the examination, a large, soft, mobile mass adjacent to the wrist on the medial aspect of his hand was identified. This was presumed to be a lipoma. However, the histopathology report revealed a benign angioleiomyoma measuring 3.2 cm, which had been completely excised during the surgery. This case report highlights the importance of considering angioleiomyoma in the differential diagnosis of soft tissue masses and the utility of US-FNA and USG-CNB in diagnosing these tumors.

## Introduction

Angioleiomyoma is a frequently painful, benign, solitary, subcutaneous, or deep dermal tumor made up of mature smooth muscle bundles surrounding and intersecting blood vessels. It is a rare tumor that is more common in middle-aged women; it tends to present in the lower extremities, and it rarely appears in the hand or fingers. While these tumors are typically smaller than 2 cm, they can grow as large as 6 cm [1,2].

The diagnosis of angioleiomyoma in the hand is often initially overlooked and can be difficult to distinguish from other types of tumors. However, it is essential to consider angioleiomyoma as a potential diagnosis, particularly when patients present with a painless mass in the hand or upper extremity. The treatment for angioleiomyoma is typically marginal excision, and the prognosis is excellent as these tumors are benign.

## Case presentation

Case one

The patient was a 41-year-old right-handed woman who visited the clinic with a two-year history of a left thumb mass. She initially suspected it was a ganglion cyst due to its small size. However, a year ago, a surgeon attempted to remove the mass but aborted the procedure due to the mass's extent, and no pathology was sent. Since then, the mass had grown significantly in size. No personal or family history of cancer existed except for hypertension, obesity, and hyperlipidemia. The patient did not experience any numbness, tingling, or pain during the visit.

During the examination, a 6x6 cm irregular mass was observed on the dorsal aspect of the left thumb, overlying the first metacarpal. The mass extended circumferentially around the base of the thumb and into the thenar eminence. However, the patient was able to actively flex, extend, and oppose the thumb to her small finger. There was no tenderness upon palpation of the mass, and the thumb exhibited good perfusion.

The patient presented with an outside X-ray and magnetic resonance imaging (MRI) of her left hand. The X-ray revealed a destructive lesion involving the first webspace with infiltration of the first metacarpal. Associated soft tissue swelling was noted in the area of the mass, along with internal calcifications within the soft tissue mass circumferentially around the first metacarpal. The MRI showed a soft tissue mass in the thenar eminence of the hand that measured 6.3 x 4.3 x 5.5 cm. The mass disrupted the cortex of the thumb metacarpal. An associated infiltrative bony lesion measured 2.5 cm and was located in the bone marrow. The soft tissues between the extensor pollicis longus tendon and the first metacarpal were infiltrated, and the lesion invaded the interosseous musculature, the first metacarpal, and the flexor and abductor pollicis tendons.

**Figure 1:**
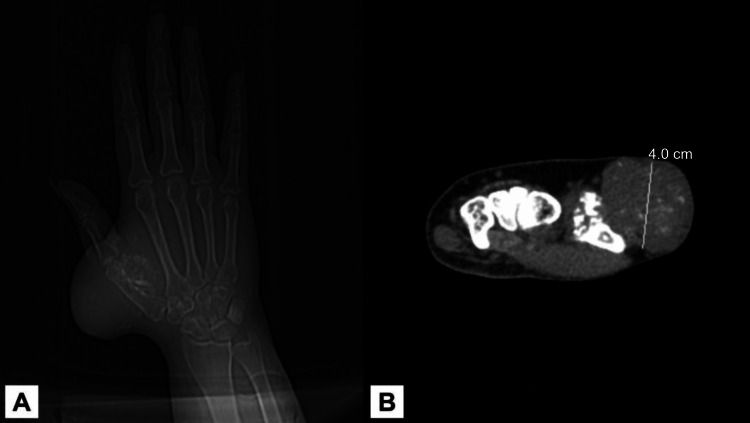
Radiological imaging findings (A) The radiograph of the left hand reveals the presence of a soft tissue mass with calcification; (B) the CT scan of the left hand indicates the existence of a lobulated mass that measures 4.0 cm in its largest anterior-posterior (AP) dimension. This mass extends into the webspace and is adjacent to the first metacarpal. The mass involves the first dorsal interosseous muscle and exhibits calcifications.

Based on the imaging studies, the mass was initially suspected to be a sarcoma, and surgical intervention, potentially including amputation of the thumb and index finger rays, was considered. A computed tomography (CT) scan of the chest and a computed tomography angiography (CTA) scan of the left hand were ordered, along with lab work. The CT scan of the left hand showed a lobulated mass measuring 4.0 x 6.0 x 5.5 cm in greatest anterior-posterior (AP), transverse, and craniocaudal dimensions, respectively, at the dorsal aspect of the first metacarpal, extending into the webspace and adjacent to the first metacarpal. This mass was predominantly located in the soft tissues; however, there was an osseous extension into the dorsal cortex of the mid-first metacarpal. The mass involved the first dorsal interosseous muscle and presented with ring and arc calcifications (Figure 1). The CTA of the left thumb showed that the axillary, brachial, radial, and ulnar arteries were widely patent. The radial artery lay at the medial aspect of the mass. The mass had marked hyperenhancement on CTA, with prominent venous collateral vascularity seen along the mass's dorsal, volar, and distal aspects. The mid-radial aspect of the mass had the least surrounding venous vascularity.

**Figure 2:**
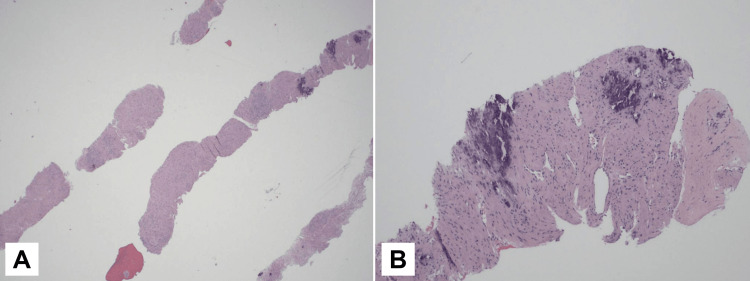
Histopathological features of the percutaneous image-guided core biopsy of the lesion (A) Hematoxylin-eosin (HE)-stained section of the core biopsy at a magnification of 20x; (B) higher magnification of the core biopsy, showing proliferation of bland spindle cells arranged in intersecting fascicles around the thin-walled vessels in a collagenous background with focal calcification. The spindle cells have elongated nuclei and tapering eosinophilic cytoplasm (HE, 100x).

**Figure 3:**
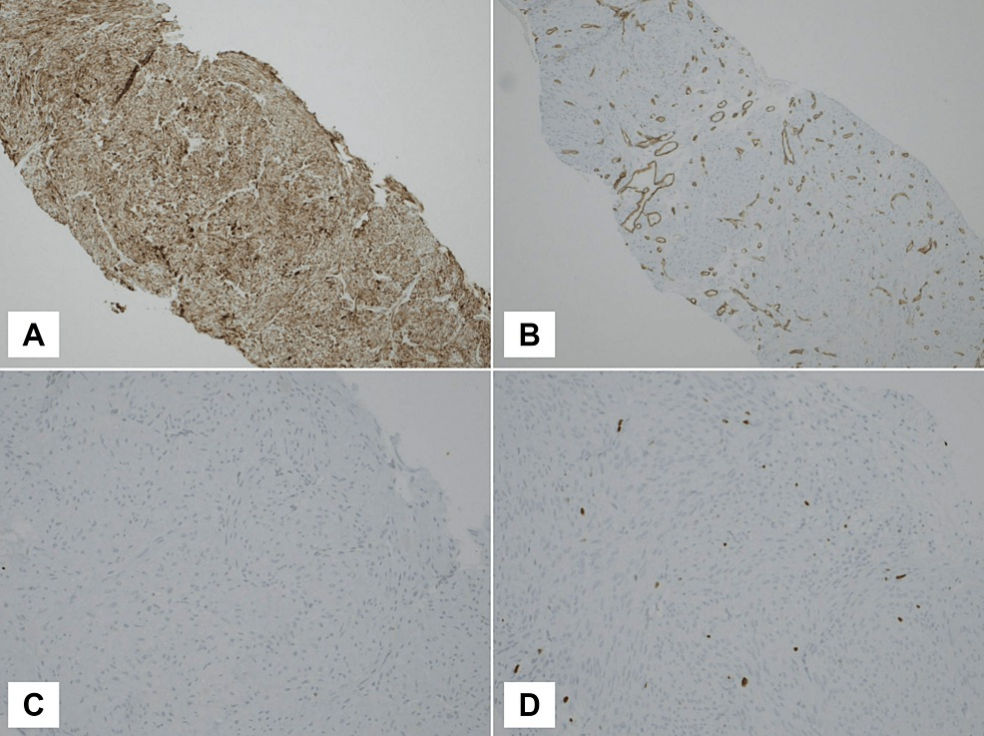
Immunohistochemistry staining of the core biopsy of angioleiomyoma (A) Smooth muscle myosin heavy chain (SMMHC) positive staining indicating the presence of smooth muscle cells in the tumor tissue (100x); (B) CD34 immunostaining highlighting blood vessels (100x); (C) S100 negative staining indicating the absence of neural cells (200x); (D) Ki67 staining showing low proliferation of the tumor cells (200x).

The patient underwent a percutaneous image-guided fine-needle aspiration (US-FNA) and an ultrasound-guided core-needle biopsy (USG-CNB) of the left-hand mass. The US-FNA reported rare benign spindle cells, a bloody background, with few neutrophils and lymphocytes, and no evidence of malignancy. The final diagnosis from the USG-CNB was angioleiomyoma, which was also confirmed by a second opinion. Tumor cells were positive for smooth muscle myosin heavy chain (SMMHC) and negative for S100 and SOX10. The endothelial cells of blood vessels were positive for CD34 (Figures 2, 3).

Afterward, the mass was surgically excised at a different medical facility. The excised surgical specimen included one segment of skin with attached subcutaneous tissue measuring 6.7 x 4.6 x 4 cm, as well as one separate segment of bony tissue measuring 5 cm in the largest dimension and another separate segment of gray-white tissue measuring 2.5 cm in the largest dimension. Upon sectioning, the tissue was found to contain a fleshy tumor with some calcification. The final diagnosis of angioleiomyoma was confirmed, which was consistent with the earlier USG-CNB.

Case two

A 63-year-old man with hypertension and end-stage renal disease presented for consultation for dialysis access creation. He was mapped to have a very suitable cephalic vein in the wrist. A large, soft, mobile mass adjacent to the wrist on the medial aspect of his hand was identified during the examination. After discussion with the patient, a decision was made to resect the mass, which was presumed to be a lipoma, at the same time the arteriovenous (AV) fistula was being created. The patient consented to both procedures.

The patient was taken to the operating room, and a 2 cm incision was made over the medial aspect of the thumb. The subcutaneous tissue was dissected, and the mass was circumferentially dissected, gently retracted with an Allis clamp, and freed from its attachments. The surgery was completed without complications, and the mass was sent to pathology for identification. The radiocephalic fistula was created with a robust thrill in the cephalic vein in the arm.

**Figure 4:**
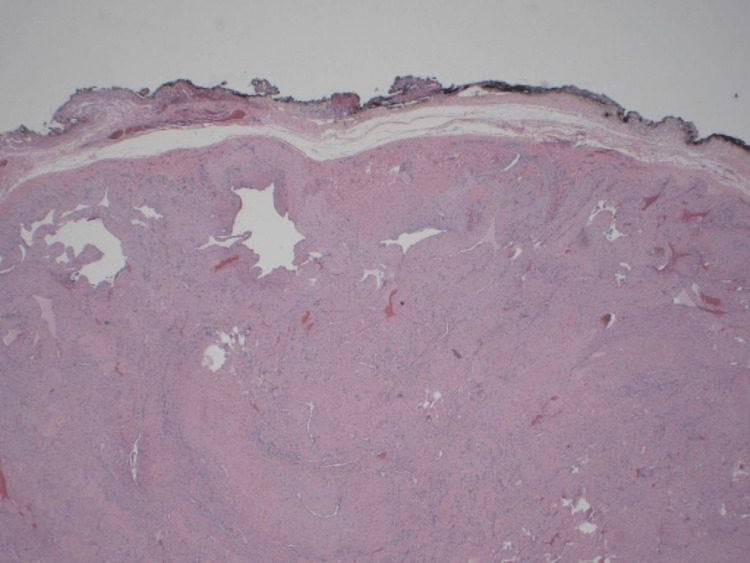
Histopathologic examination of the angioleiomyoma: low-power view of the tumor margin (HE, 40x) HE: Hematoxylin-eosin

**Figure 5:**
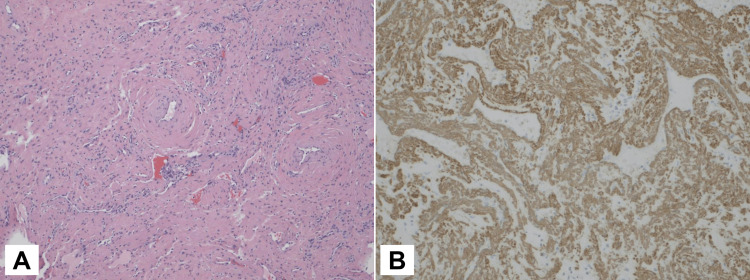
Histopathologic examination of the angioleiomyoma (A) Hematoxylin-eosin (HE) stain of tumor tissue showing spindle cells and various-sized blood vessels with different vessel wall thicknesses (100x); (B) smooth muscle actin (SMA) positive staining in tumor cells (200x)

The histopathology report revealed a benign angioleiomyoma measuring 3.2 cm, which had been completely excised. Microscopic examination revealed a well-circumscribed tumor composed of spindle-shaped cells with interspersed various-sized blood vessels with different wall thicknesses. No significant increase in mitoses or necrosis was seen. The tumor cells were positive for smooth muscle actin (SMA), consistent with angioleiomyoma (Figures 4, 5).

## Discussion

Angioleiomyomas are slow-growing, painful tumors commonly occurring in the lower extremities and are more common in women. These tumors are often misdiagnosed and can be mistaken for a ganglion cyst, inclusion cyst, lipoma, fibroma, synovial sarcoma, other low-grade soft tissue sarcomas, haemangioma, and neurogenic tumor. Histopathology is necessary to establish the correct diagnosis. Angioleiomyoma is a benign tumor of smooth muscle origin characterized by a proliferation of well-differentiated smooth muscle cells with abundant vascularization [1-3].

Angioleiomyomas in the hand are rare, and they are even more uncommon in the fingers. On physical examination, they appear as a subcutaneous oval or round mass with a maximum diameter ranging from 0.7 cm to 6.0 cm. Imaging findings are usually nonspecific, and on CT, angioleiomyomas may appear as a well-defined and otherwise unspecific cutaneous or subcutaneous soft tissue density mass, sometimes with calcifications. The diagnosis requires an excisional biopsy with confirmatory immunohistochemical evaluation [4-6].

On microscopic examination, angioleiomyomas are characterized by a well-circumscribed, non-encapsulated mass of smooth muscle cells arranged in fascicles or bundles. Blood vessels, which are the defining feature of angioleiomyomas, are often seen within the tumor mass and may appear dilated and surrounded by smooth muscle cells. Immunohistochemical evaluation of angioleiomyoma typically shows positive staining for SMMHC, SMA, desmin, and h-caldesmon, which are markers of smooth muscle cells. In addition, angioleiomyomas often show positive staining for CD34, a marker of endothelial cells commonly expressed in the blood vessels within the tumor [2,5,6]. These findings can help distinguish angioleiomyomas from other types of soft tissue tumors that may have similar features on microscopic examination. In our cases, the preoperative diagnoses were ganglion cysts, soft tissue sarcomas, and lipomas.

Ganglion cysts are common, benign lesions often occurring on the hands and wrists. Histopathologic examination typically reveals a cystic structure filled with clear or mucinous fluid, lined by synovial cells, and surrounded by fibrous tissue. Lipomas are the most common soft tissue tumors; they are typically slow-growing, painless, and well-circumscribed. Histologically, lipomas are composed of mature adipocytes and fibrous tissue. Synovial sarcomas are malignant tumors that often occur in the extremities and consist of spindle-shaped cells arranged in a biphasic pattern with glandular or epithelial differentiation areas. Low-grade soft tissue sarcomas, such as leiomyosarcoma and myxoid liposarcoma, are malignant tumors that can be difficult to distinguish from benign tumors on imaging and gross examination. Histopathologically, both are spindle cell tumors with distinct morphologies. Immunohistochemistry may show positive staining for specific markers, such as SMA for leiomyosarcoma and S100 for myxoid liposarcoma [2].

The mechanism of development of angioleiomyomas remains unclear. While classified as vascular hamartomas, these tumors' etiology has also been linked to mechanical and hormonal causes [7]. We presented two cases of angioleiomyomas of the hand that were initially misdiagnosed but later identified as angioleiomyomas through histopathology. The importance of obtaining a biopsy for histopathology was highlighted in both cases. Treatment for angioleiomyoma involves complete surgical excision, and recurrence is uncommon.

## Conclusions

In conclusion, our case series highlights the importance of thorough evaluation and histopathological examination in diagnosing hand tumors. Although rare, angioleiomyoma should be considered in the differential diagnosis of painless, subcutaneous, well-circumscribed tumors of the hand and finger. A histopathological analysis is necessary for an accurate diagnosis and appropriate management. The treatment involves complete surgical excision, usually curative, with minimal recurrence.
